# Analysis of screening outcomes and factors influencing compliance among community-based lung cancer high-risk population in Nanchang, China, 2018-2020

**DOI:** 10.3389/fonc.2024.1339036

**Published:** 2024-02-09

**Authors:** Fanfan Zeng, Xiaobo Wang, Chengman Wang, Yu Zhang, Denggang Fu, Xin Wang

**Affiliations:** ^1^ Jiangxi Provincial Key Laboratory of Preventive Medicine, School of Public Health, Nanchang University, Nanchang, Jiangxi, China; ^2^ Jiangxi Provincial Key Laboratory of Systems Biomedicine, Jiujiang University, Jiujiang, Jiangxi, China; ^3^ Cancer Center, Jiangxi Provincial Tumor Hospital, Nanchang, Jiangxi, China; ^4^ College of Medicine, Medical University of South Carolina, Charleston, SC, United States

**Keywords:** lung cancer, high-risk population screening, low dose spiral CT, compliance, Jiangxi Province

## Abstract

**Objective:**

To investigate the screening results and compliance of low-dose computed tomography (LDCT) screening among the high-risk lung cancer populations in Jiangxi Province from 2018 to 2020, and to explore the related influencing factors of compliance.

**Methods:**

From November 2018 to October 2020, permanent residents in Nanchang City were selected and their demographic data and lung cancer risk factor data were collected to screen high-risk groups, and LDCT screening was performed on high-risk groups with diagnostic reports by 2 chief physicians. Descriptive analysis method was used to analyze the basic information of screening, screening results and screening compliance. χ2 and logistic regression test were used to conduct single and multi-factor analysis of screening compliance.

**Results:**

A total of 26,588 people participated in this screening, of which 34.4% (n=9,139) were at high risk of lung cancer, 3,773 participants were completed LDCT screening, and the screening compliance rate was 41.3%. Screening results showed that 389 participants were positive for suspected pulmonary tumor or lung nodules, the screening positive rate of 10.3%. The logistic multivariable results of screening compliance showed that the compliance was better in males, those who quit smoking, those with chronic respiratory diseases and family history of cancer, and those who have primary education, those with a history of occupational harmful exposure had a poor compliance.

**Conclusion:**

Compliance with lung cancer screening in Jiangxi Province, China still needs to be improved, and gender, education level, harmful occupational exposure, smoking, chronic respiratory diseases, and family history of tumors cancer play an important role on screening compliance.

## 1 Introduction

Lung cancer is the second most common diagnosed cancer and the leading cause of cancer death in 2020, accounting for 20% of cancer-related death (1). The National Cancer Center’s latest statistics indicated that there were approximately 2,413,500 cancer-related deaths in China in 2016. Lung cancer was the most common cause of cancer deaths in both sexes, accounting for 22.92% (202,300) of the total number of cancer deaths in females and 29.71% (454,700) in males (2). Lung cancer has long latency without explicit symptoms which lead to approximately 70% of patients are diagnosed at advanced stage with a poor prognosis (3, 4). The advances in cancer diagnosis and treatment improve patients’ outcomes, while the patients who are diagnosed at advanced stage have a low 5-year survival rate (5). The most effective preventive strategy for lung cancer is to diagnose lung cancer patients at early stage which allows for timely intervention to improve the life quality of patients and extend their survival rate (6). Therefore, as the biggest developing country, it is imperative to implement lung cancer screening program to enrolls the communities that are potentially exposed at risk environment. LDCT screening provides an effective method for the early detection of lung cancer. Increasing studies have shown that LDCT can reduce the overall mortality of lung cancer by 20% compared with chest X-ray (7). The effectiveness of screening work largely depends on the compliance of the population on the screening, and a lower compliance could hinder the implementation of screening program to move forward (8). Therefore, it is of great significance to identify the factors affecting screening compliance which contributes to improve the early diagnosis and treatment of lung cancer, and to prolong the survival. This study intends to analyze the potential factors affecting screening compliance to participate in LDCT screening in the Urban Cancer Screening and Early Detection and Treatment Program, providing data support for further optimization of lung cancer screening and improvement of screening compliance.

## 2 Materials and methods

### 2.1 Participants

The study subjects were from the Urban Lung Cancer Early Detection and Treatment Program, and residents who met the inclusion criteria were recruited by community service center based on the principle of voluntariness for survey and assessment. Inclusion criteria: 1. household residents of Nanchang City who have lived in the city for more than 3 years; 2. aged 40-74 years old; 3. signed written informed consent; 4. have full behavioral ability. Exclusion criteria: 1. previous history of tumor; 2. suffering from serious heart, brain, lung disease or renal dysfunction.

### 2.2 Investigation contents and high-risk assessment methods

The survey used a cancer risk assessment questionnaire, which included information on socio-demographic data, lifestyle behavioral habits, past history of disease, and family history of tumors. Survey respondents were surveyed by uniformly trained surveyors using a face-to-face survey format. The questionnaire information was then entered into the National Cancer Prevention and Control Platform’s Early Diagnosis and Early Treatment Risk Assessment Database by a specially designed database to assess the lung cancer risk group. The database is based on the Harvard Cancer Risk Index, which is a comprehensive evaluation system of individual cancer risk that is suitable for Chinese population and has been discussed and approved by a multidisciplinary panel of experts (9). For those who are confirmed to be high-risk groups, we recommend them to go to Jiangxi Cancer Hospital for free LDCT screening, and the results of the screening will be assessed by at least two chief physicians for diagnosis.

### 2.3 Result judgment and definition

Positive nodules: non-solid nodules ≥8 mm; solid nodules or partially solid nodules ≥5 mm.2. Suspected lung cancer: determined by a senior physician based on imaging data and clinicopathologic diagnosis.3. The index for evaluating screening adherence was the screening participation rate, which was defined as screening participation rate = number of people who participated in LDCT screening/number of people who were at high risk of lung cancer screening*100% (8). 4. Smokers were defined as smoking more than one cigarette per day for more than 6 months. 5. People who drink at least once pre week for one year were defined as drinkers. 6. Physical exercise was defined as an average of more than 3 times per week for more than 30 minutes. 7. Harmful occupational exposure was defined as cumulative exposure to hazardous substances for more than 1 year.

### 2.4 Statistical analysis

SPSS25.0 software was used for data processing and analysis. Descriptive analysis method was used to analyze the basic information of screening, screening results and screening compliance. χ2 test and logistic regression test were used to conduct single and multi-factor analysis of screening compliance. Two-sided P-values < 0.05 was considered as statistically significant.

## 3 Result

### 3.1 Screening basic information

A total of 26,588 participants were enrolled in this study as shown in **Table 1**, of which 34.4% (9,139) were identified as high-risk for lung cancer and 65.6% (17,449) were excluded as non-high-risk for lung cancer. The average age of high-risk participants was 63.670 ± 6.597 years, and that of non-high-risk enrollment was 61.510 ± 8.692 years. Among these high-risk participants, there were 3,603 males, accounting for 39.4% and women (5,536) account for 60.6%. There were 3,541 smokers fall in the high-risk group while 9.6% (1,673) smokers were out of high-risk group.

**Table 1:** Basic information on high-risk and non-risk groups for lung cancer.

basic characteristic	high-risk group (n=9139)	non-high-risk group (n=17449)	*P* value
Age (years, $\bar{\text{x}}$ ± s)	63.670 ± 6.597	61.510 ± 8.692	<0.01
BMI (kg/m^2^, $\bar{\text{x}}$ ± s)	23.942 ± 4.910	23.749 ± 5.991	0.733
Gender			<0.01
Male	3603 (39.4)	6166 (35.3)	
Female	5536 (60.6)	11283 (64.7)	
Educational level			<0.01
Primary and under	1071 (11.7)	1930 (11.1)	
Junior High School	3030 (33.2)	5825 (33.4)	
High School/Middle College/Technical College	2765 (30.3)	5284 (30.3)	
Specialty	1697 (18.6)	3087 (17.7)	
Undergraduate	376 (4.1)	898 (5.1)	
Postgraduate or above	200 (2.2)	425 (2.4)	
Marital status			<0.01
Unmarried	41 (0.4)	94 (0.5)	
Married	8728 (95.5)	16561 (94.9)	
Remarried	63 (0.7)	181 (1.0)	
Divorced	45 (0.5)	133 (0.8)	
Widowed	261 (2.9)	478 (2.7)	
Harmful occupational exposure ^a^			<0.01
No	6396 (70.0)	16836 (96.5)	
Yes	2743 (30.0)	613 (3.5)	
Smoking			<0.01
Non-smoking	5301 (58.0)	15396 (88.2)	
Smoking	3541 (38.7)	1673 (9.6)	
Quit	297 (3.2)	380 (2.2)	
Drinking			<0.01
No	6383 (69.8)	15678 (89.9)	
Yes	2756 (30.2)	1771 (10.1)	
Physical exercise			<0.01
No	5459 (59.7)	7763 (44.5)	
Yes	3680 (40.3)	9686 (55.5)	
Chronic respiratory diseases ^b^			<0.01
No	5134 (56.2)	16072 (92.1)	
Yes	4005 (43.8)	1377 (7.9)	
Family history of tumors			<0.01
No	4658 (51.0)	14124 (80.9)	
Yes	4481 (49.0)	3325 (19.1)	

^a^ Hazardous occupational exposure includes exposure to asbestos, radon, beryllium, uranium, benzene and coal tar, etc., which have been clearly identified as carcinogenic; ^b^ Chronic respiratory diseases include tuberculosis, chronic bronchitis, emphysema, asthma, bronchiectasis, silicosis or pneumoconiosis.

### 3.2 Screening results

Among 3,773 people were screened by LDCT, 355 cases showed positive lung nodules, accounting for 9.4%; 34 cases of suspected lung cancer, accounting for 0.9%; 1,343 cases exhibited inflammation in the lungs or other diseases of the lungs, accounting for 35.6%. A total of 2,041 cases showed no abnormality measured using CT screening. accounting for 54.1%. The positive rate of suspected lung cancer or positive lung nodules was 10.3%, of which 10.9% (197/1808) were male and 9.8% (192/1965) were female; of which 9.8% (120/1224) were in the 50-59 years, 10.0% (177/1773) in the 60-69 years, and the positive rate of participants with greater than 70 years was 11.3% (92/816).

### 3.3 One-way analysis of compliance

People who completed LDCT screening in high-risk lung cancer groups were included in compliance analysis, and those who did not complete LDCT screening were included in non-compliance group. As displayed in **Table 2**, 3,773 who completed LDCT screening have a screening compliance rate of 41.3%. The gender, age, education level, marital status, occupational exposure to harmful substances, smoking, drinking, regular physical exercise, chronic respiratory diseases and family history of cancer (P ≤ 0.05) showed statistically difference in screening compliance vs non-compliance groups.

**Table 2:** Results of one-way analysis of factors influencing lung cancer screening compliance.

Factor	Number of non-adherent groups (n=5366)	Number of adherent groups (n=3773)	Compliance rate (%)	χ^2^	*P* value
Gender				194.177	<0.01
Male	1795	1808	50.2		
Female	3571	1965	35.5		
Age groups (years)				8.193	0.017
50~	1772	1224	40.9		
60~	2320	1733	42.8		
≥70	1274	816	39.0		
BMI groups				0.471	0.925
≤18.5	138	103	42.7		
18.5~	2814	1969	41.2		
24~	1939	1356	41.2		
≥28	475	345	42.1		
Educational level				105.036	<0.01
Primary and under	770	301	28.1		
Junior High School	1774	1256	41.5		
High School/Middle College/Technical College	1581	1184	42.8		
Specialty	900	797	47.0		
Undergraduate	213	163	43.4		
Postgraduate or above	128	72	36.0		
Marital status				27.754	<0.01
Unmarried	21	20	48.8		
Married	5108	3620	41.5		
Remarried	29	34	54.0		
Divorced	20	25	55.6		
Widowed	188	74	28.2		
Harmful occupational exposure				4.659	0.031
No	3802	2594	40.6		
Yes	1564	1179	43.0		
Smoking				182.603	<0.01
Non-smoking	3509	1940	35.6		
Smoking	1792	1749	49.4		
Quit	65	84	56.4		
Drinking				52.959	<0.01
No	3905	2478	38.8		
Yes	1461	1295	47.0		
Physical exercise				11.794	<0.01
No	3126	2333	42.7		
Yes	2240	1440	39.1		
Chronic respiratory diseases				94.942	<0.01
No	3242	1892	36.9		
Yes	2124	1881	47.0		
Family history of tumors				115.652	<0.01
No	2988	1670	35.9		
Yes	2378	2103	46.9		

### 3.4 Logistic multi-factor analysis

A logistic multivariable analysis was conducted with the screening adherence subgroups of lung cancer high-risk groups as the dependent variable, and gender, age, education level, marital status, occupational exposure to harmful substances, smoking, drinking, regular participation in physical exercise, chronic respiratory disease and family history of cancer as independent variables (**Table 3**). We found that screening compliance was worse in women (OR=0.623,95%CI: 0.532-0.728) as compared with men. The compliance of people aged 60 to 70 years was better than that of people aged over 70 years (OR=1.137,95%CI: 1.016 to 1.273). Compared with the population with education level in primary school or below, the population with education level in junior high school (OR=1.412,95%CI: 1.206~1.653), senior high school (OR=1.393,95%CI: 1.186~1.635)/middle college/technical college (OR=1.587,95%CI: 1.335~1.886) had better compliance; The compliance of the population with harmful occupational exposure was lower than that of the population without harmful occupational exposure (OR=0.842,95%CI: 0.761-0.932). The compliance of quitter was better than that of non-smokers and smokers (OR=0.603,95%CI: 0.422~0.863), (OR=0.660,95%CI: 0.472~0.924). People with chronic respiratory disease had better screening compliance than those without chronic respiratory disease (OR=1.280,95%CI: 1.161~1.410). People with a family history of cancer had better compliance (OR=1.457,95%CI: 1.326~1.601).

**Table 3:** Results of logistic multivariable analysis of factors influencing compliance with lung cancer screening.

Factor	*P* value	*OR*	*OR95%CI*
Gender			
Male		1.000	
Female	<0.01	0.623	(0.532~0.728)
Age groups (years)			
50~	0.227	1.078	(0.954~1.217)
60~	0.025	1.137	(1.016~1.273)
≥70		1.000	
Educational level			
Primary and under		1.000	
Junior High School	<0.01	1.412	(1.206~1.653)
High School/Middle College/Technical College	<0.01	1.393	(1.186~1.635)
Specialty	<0.01	1.587	(1.335~1.886)
Undergraduate	0.074	1.261	(0.978~1.626)
Postgraduate or above	0.283	0.835	(0.600~1.161)
Harmful occupational exposure			
No		1.000	
Yes	<0.01	0.842	(0.761~0.932)
Smoking			
Non-smoking	<0.01	0.603	(0.422~0.863)
Smoking	0.016	0.660	(0.472~0.924)
Quit		1.000	
Chronic respiratory diseases			
No		1.000	
Yes	<0.01	1.280	(1.161~1.410)
Family history of tumors			
No		1.000	
Yes	<0.01	1.457	(1.326~1.601)

## 4 Discussions

Lung cancer is the most common and deadly tumor in the world, and the largest public health challenge posed by pulmonary tumor is the poor prognosis in the advanced stage. Studies have found that the prognosis of patients with lung cancer is closely related to disease stage. The five-year survival rate of patients with early stage lung cancer is 60%, and that of patients with middle and advanced stage lung cancer strikely decrease to 5%-40% (10). Therefore, the implementation of lung cancer screening to detect patients with early stage lung cancer is one of the main steps needed to reduce lung cancer-related deaths and improve survival. The study analyzed the screening data from the urban cancer early detection and early treatment project of Jiangxi Province from 2018 to 2020. The screening involved 26,588 participants in 8 administrative regions of Nanchang City. The results showed that there were 9,139 high-risk groups of lung cancer, among which 3,773 completed LDCT screening, and the screening compliance rate was 41.3%, which is higher than the overall participation rate of 34.8% (8) in Zhejiang, Anhui and Liaoning provinces, 37.5% (11) in Henan Province and 37.10% (12) in Beijing. Among 3,773 participants in LDCT screening, 355 were positive for nodules, 34 were suspected of lung cancer, and the positive rate of suspected lung cancer or lung nodules was 10.3%.

This study also further analyzed the influencing factors of screening compliance among high-risk groups of lung cancer, and found that gender, educational level, harmful occupational exposure, smoking, chronic respiratory diseases and family history of cancer had important effects on screening compliance. The results show that the compliance of men is better than that of women, which may be related to smoking. The majority of men had smoking history, which has been demonstrated to be the risk factor of a variety of lung diseases (13). These smokers are willing to take care of their lungs condition, and LDCT can provide a preliminary detection of the lungs, so the compliance of those men may be better than that of women. Among different age groups, the compliance of people aged 60-70 is better than that of people aged over 70, which is consistent with the results of other studies (8, 14). Participants with greater than 70 years might have basic diseases such as hypertension and diabetes, which made them in poor physical condition and inconvenient to participate, weaken their enthusiasm to be involved. Some elders have unfavorable life condition or live far from the screening center which decrease their willingness. The low compliance of people with primary school education may be due to their low health awareness and poor knowledge of lung cancer, and failing to recognize the importance of screening for early detection and diagnosis, which is consistent with the results of studies in Guangzhou (15) and Hebei (16). The compliance of people exposed to occupational harmful factors is lower than that of people not exposed to occupational harmful factors, which may be due to the fact that people exposed to occupational harmful factors will arrange a regular time for physical screening, and they are relatively aware of their own conditions, so they fail to participate in screening.

Studies have shown that smoking, chronic respiratory diseases and family history of cancer are risk factors for lung cancer (17, 18). The results of the screening showed that the compliance with screening was better in quitters than in non-smokers and smokers, probably because they believed that due to not smoking, their lung condition is better, so there is no need to check their lungs; the quitters were more likely to undergo LDCT screening compared with the smokers, probably because they were gradually learning about the relationship between smoking and lung cancer, and they knew that smokers had a higher relative risk of lung cancer (19) and that they had smoked before. In addition, it is consistent with previous studies (8, 15) that people with chronic respiratory diseases and family history of tumors have better screening compliance, which may be due to the fact that people with chronic respiratory diseases are relatively more familiar with lung diseases, and it is also recommended by doctors to check their lung conditions regularly. For people with a family history of cancer, the illness of relatives makes them have more understanding of cancer, and they have a higher sense of identity for early detection of cancer by screening, so this may be the reason for the relatively good compliance of these two groups of people.

In conclusion, the compliance in this area still needs to be improved, and our relevant staff should strengthen the publicity and education work on early detection and early diagnosis and early treatment of cancer in ordinary times. Screening staff should pay attention to the factors that have an important influence on screening compliance and try to avoid them during the implementation of screening work in the future, so as to further improve screening compliance, increase the cancer detection rate, and enable patients with early stage of lung cancer to receive treatment in time, so as to improve their quality of life and prolong their survival time. Based on the results of the survey, we suggest that we focus on strengthening publicity and education for people over 70 years of age and those with elementary school education because the incidence of lung cancer in people over 70 years of age is the highest compared with the other two age groups (14, 20), but their compliance is still poor, therefore, maybe we could screen them when they regularly get prescriptions or screen the inconvenient elders at their home, and the compliance of those with elementary school education is also lower than that of those with other levels of education, therefore, it is very necessary to let them know the health hazards of lung cancer to the population, and to recognize the importance of screening for early detection, diagnosis, and treatment, so as to encourage them to actively participate in the screening, and to increase the screening compliance. However, this study still has potential limitations. Since the screening was completed in the form of investigation, the recall bias generated during the investigation was unavoidable; As the analysis is based on a local population, the generalizability of this study is limited; We only analyzed the screening compliance of high-risk group, and did not evaluate in non-high-risk group. Further improvement of the research content is warranted by including comprehensive factors, which helps to produce meaningful findings.

## Data availability statement

The raw data supporting the conclusions of this article will be made available by the authors, without undue reservation.

## Ethics statement

This study was approved by the medical ethical review committee of Jiangxi Provincial Cancer Hospital.

## Author contributions

FZ: Methodology, Validation, Writing – original draft, Data curation, Software. XBW: Data curation, Investigation, Writing – original draft. CW: Data curation, Writing – original draft. YZ: Software, Writing – original draft. DF: Writing – review & editing, Formal Analysis. XW: Writing – review & editing.
